# Correction to: Dose–response relationship of pulmonary disorders by inhalation exposure to cross-linked water-soluble acrylic acid polymers in F344 rats

**DOI:** 10.1186/s12989-022-00475-w

**Published:** 2022-05-13

**Authors:** Tomoki Takeda, Shotaro Yamano, Yuko Goto, Shigeyuki Hirai, Yusuke Furukawa, Yoshinori Kikuchi, Kyohei Misumi, Masaaki Suzuki, Kenji Takanobu, Hideki Senoh, Misae Saito, Hitomi Kondo, George Daghlian, Young-Kwon Hong, Yasuhiro Yoshimatsu, Masanori Hirashima, Yoichiro Kobashi, Kenzo Okamoto, Takumi Kishimoto, Yumi Umeda

**Affiliations:** 1 Japan Bioassay Research Center, Japan Organization of Occupational Health and Safety, Hadano, Kanagawa 257-0015 Japan; 2 Department of Surgery, Norris Comprehensive Cancer Center, Keck School of Medicine, University of Southern California, Los Angeles, CA USA; 3 Division of Pharmacology, Niigata University Graduate School of Medical and Dental Sciences, Niigata, 951-8510 Japan; 4 Department of Pathology, Tenri Hospital, Tenri, Nara 632-8552 Japan; 5 Department of Pathology, Hokkaido Chuo Rosai Hospital, Japan Organization of Occupational Health and Safety, Iwamizawa, Hokkaido 068-0004 Japan; 6 Director of Research and Training Center for AsbestosRelated Diseases, Okayama, Okayama 702-8055 Japan

## Abstract

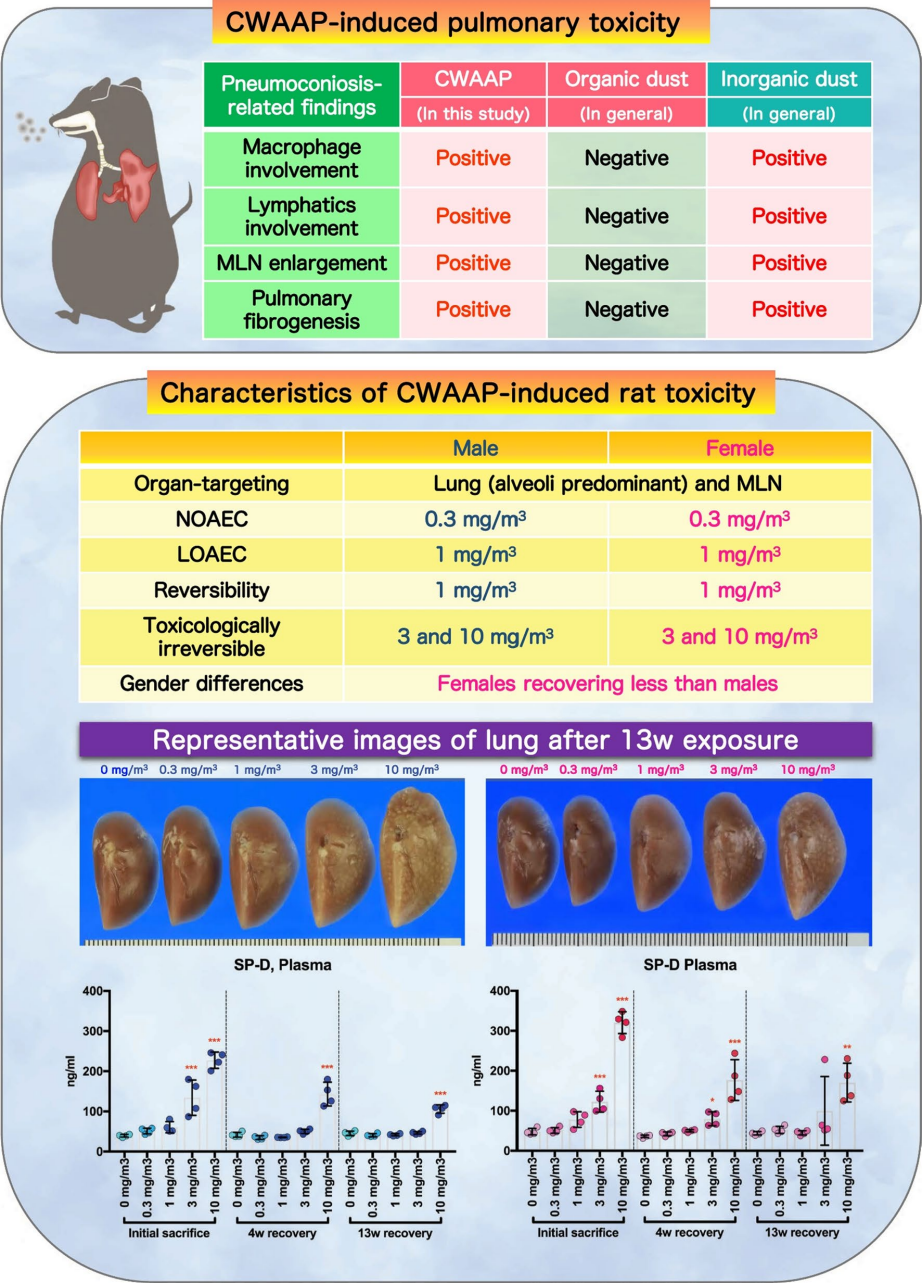

## Correction to: Particle and Fibre Toxicology (2022) 19:27 10.1186/s12989-022-00468-9

Following publication of the original article [1], the authors reported that the Graphical Abstract was missing.

The Graphical Abstract has been provided in this Correction.

The original article [1] has been updated.
