# Supplementary figures and images for: Dose–response relationship of pulmonary disorders by inhalation exposure to cross-linked water-soluble acrylic acid polymers in F344 rats
Source: Part Fibre Toxicol. 2022 Apr 8;19:27. doi: 10.1186/s12989-022-00468-9 (PMC8994297; doi:10.1186/s12989-022-00468-9)

Fig. S1

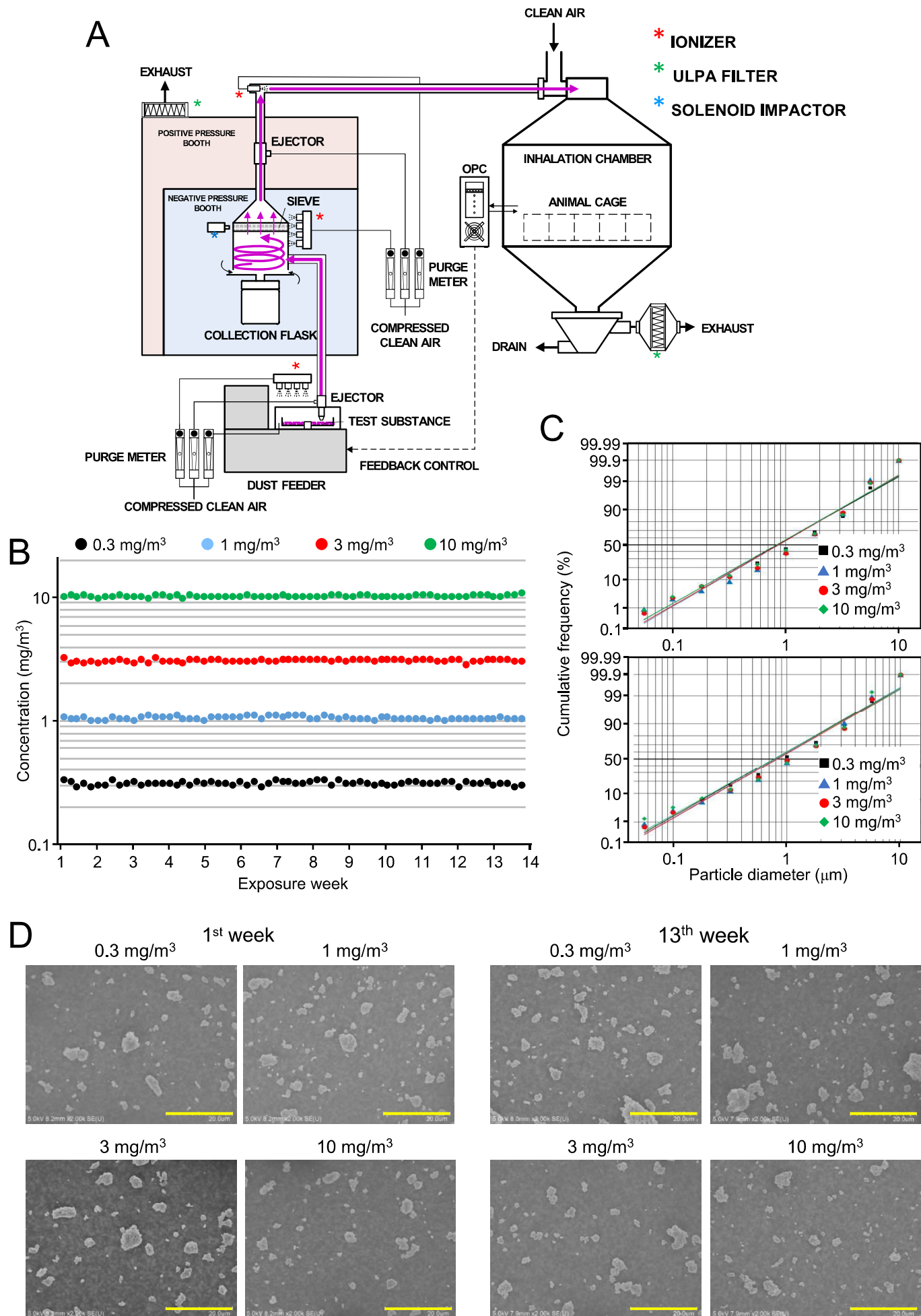

Supplement: Supplementary file 1 — Additional file 1: Fig. S1. The whole body inhalation exposure system using in this study. The whole body inhalation exposure system (A), the averaged CWAAP-A concentration in the chamber per each exposure day (B), cumulative frequency distribution graphs with logarithmic probability (C) and representative scanning electron microscope (SEM) images of the CWAAP-A particles in the chambers (D). Scale bar: 20 μm (panel D). [file 12989_2022_468_MOESM1_ESM.pdf]

Fig. S2

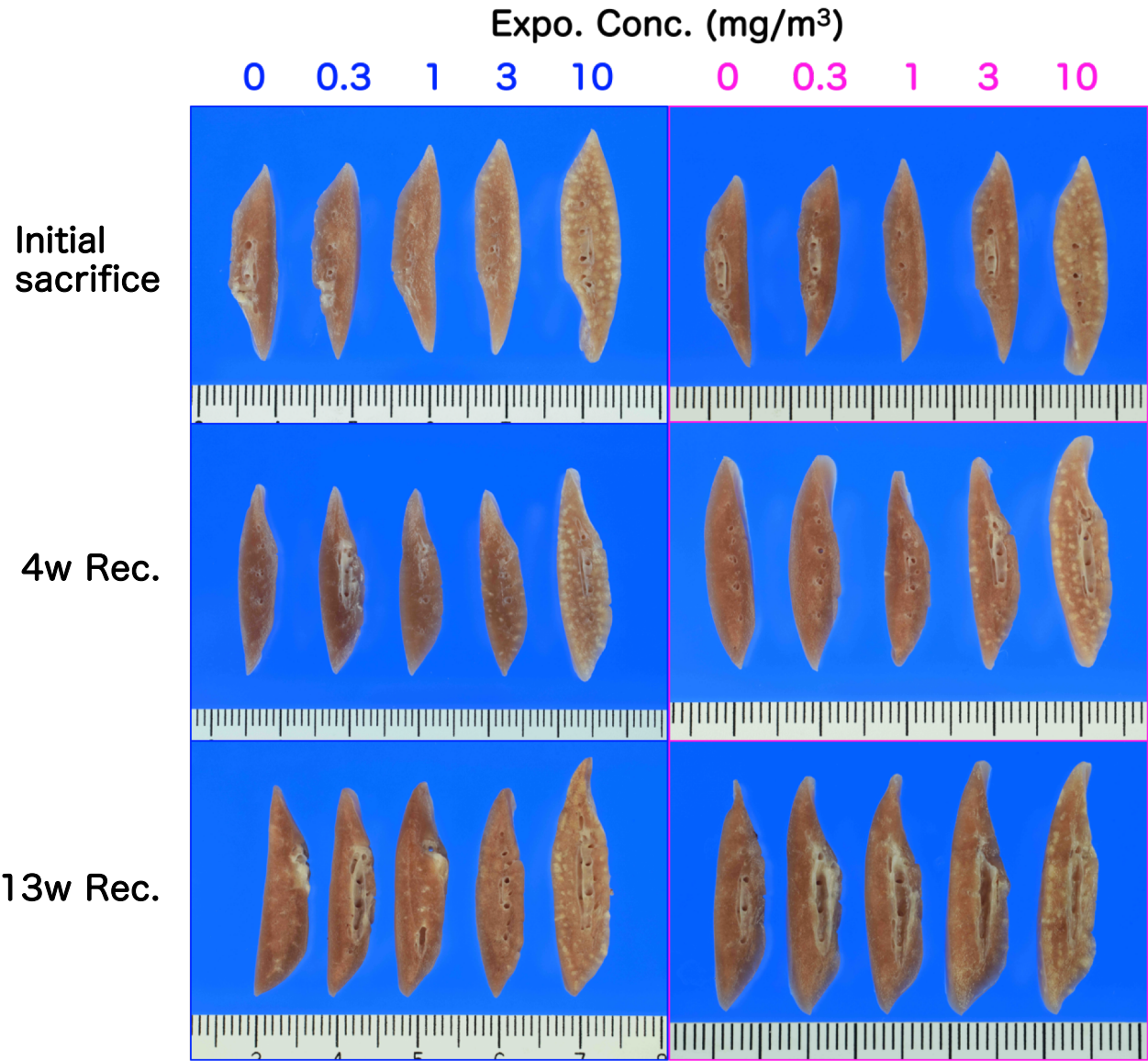

Supplement: Supplementary file 2 — Additional file 2: Fig. S2. Representative macroscopic photographs of cross-sections of rat lungs. The left and right sides represent males and females, respectively. conc concentration, expo exposure, rec recovery. [file 12989_2022_468_MOESM2_ESM.pdf]

Fig. S3

A

Prox1-EGFP rat lung  
 $\alpha$ SMA/GFP/Hematoxylin

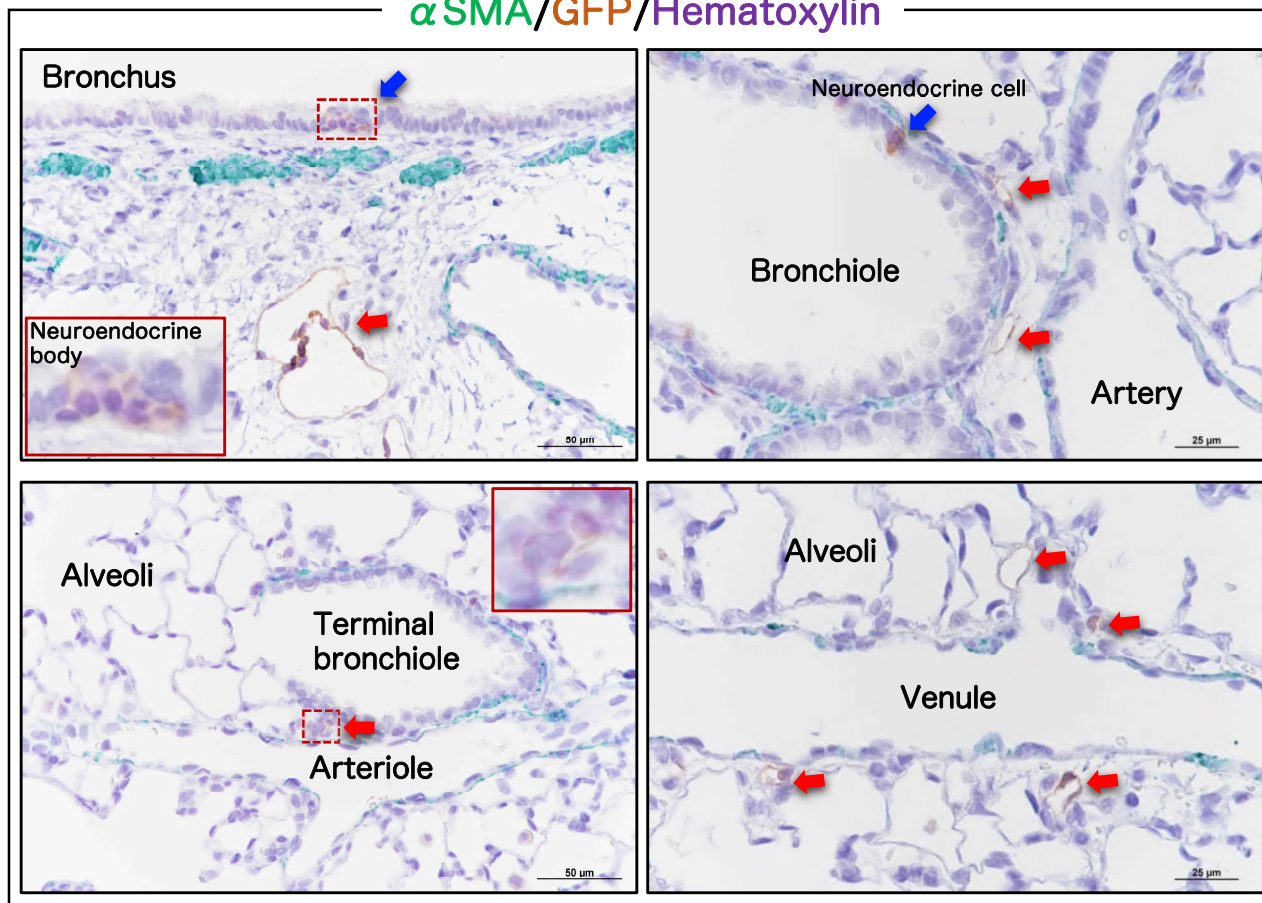

B

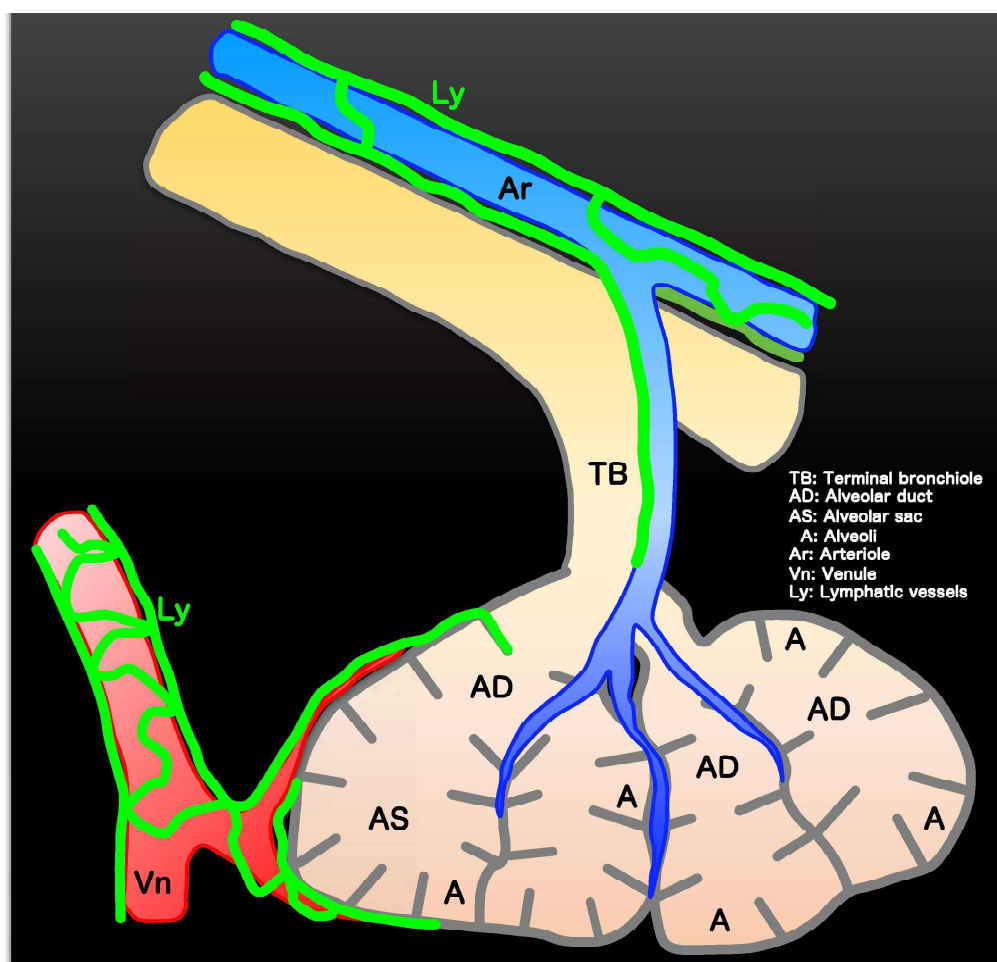

Supplement: Supplementary file 3 — Additional file 3: Fig. S3. Visualization of normal lymphatic vessels in the rat lung using Prospero homeobox protein-1 (Prox1)-enhanced green fluorescent protein (EGFP) transgenic rats [13]. (A) Lung sections from Prox1-EGFP transgenic rats were double-stained with GFP and α-smooth muscle actin (αSMA) and counterstained with hematoxylin. Lymphatic vessels (red arrows) run around the veins and arteries. Blue arrows represent neuroendocrine cells. (B) A graphical image of lymphatic vessels running (green lines) through the lungs. [file 12989_2022_468_MOESM3_ESM.pdf]

Fig. S4

A

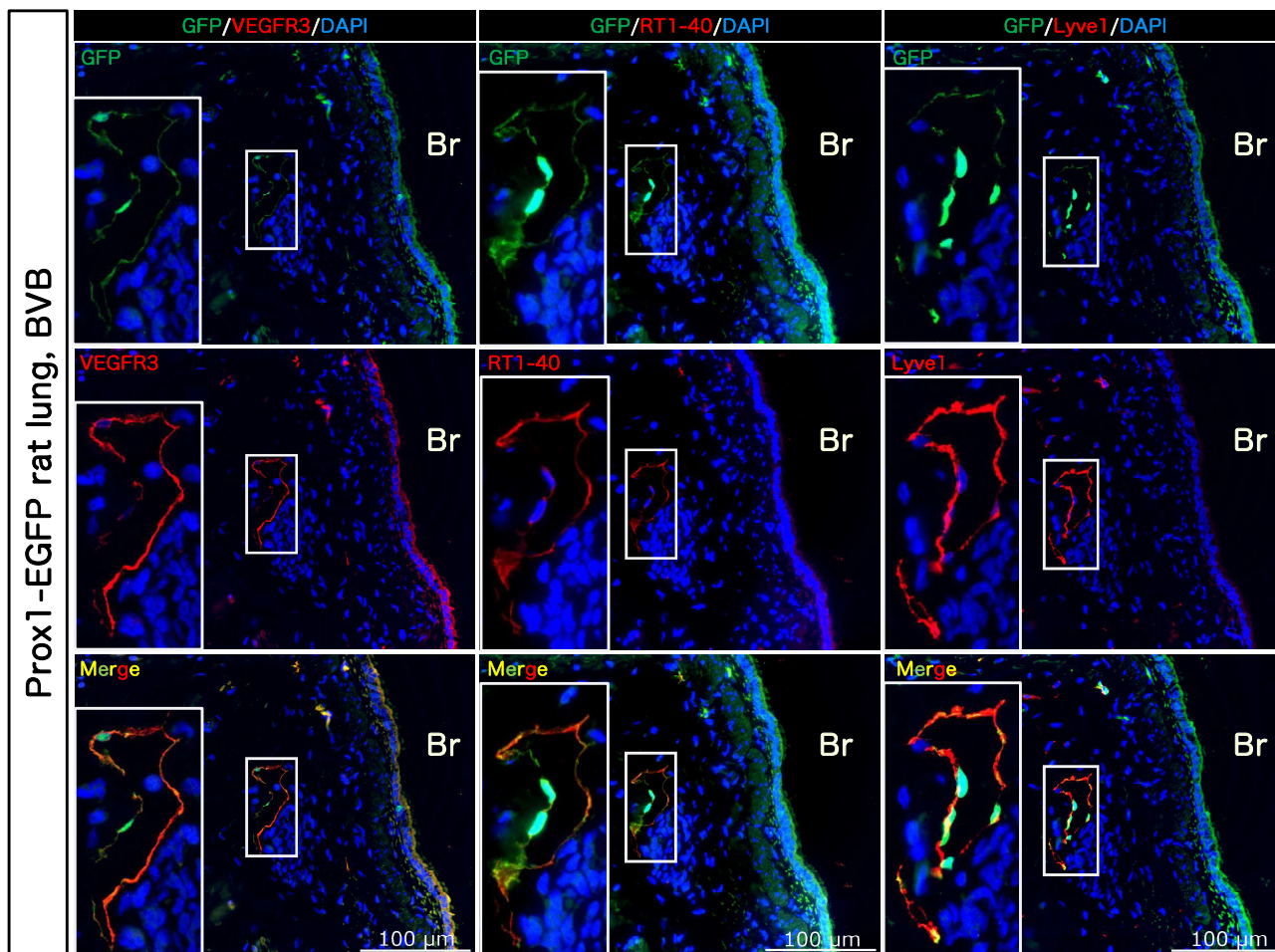

B

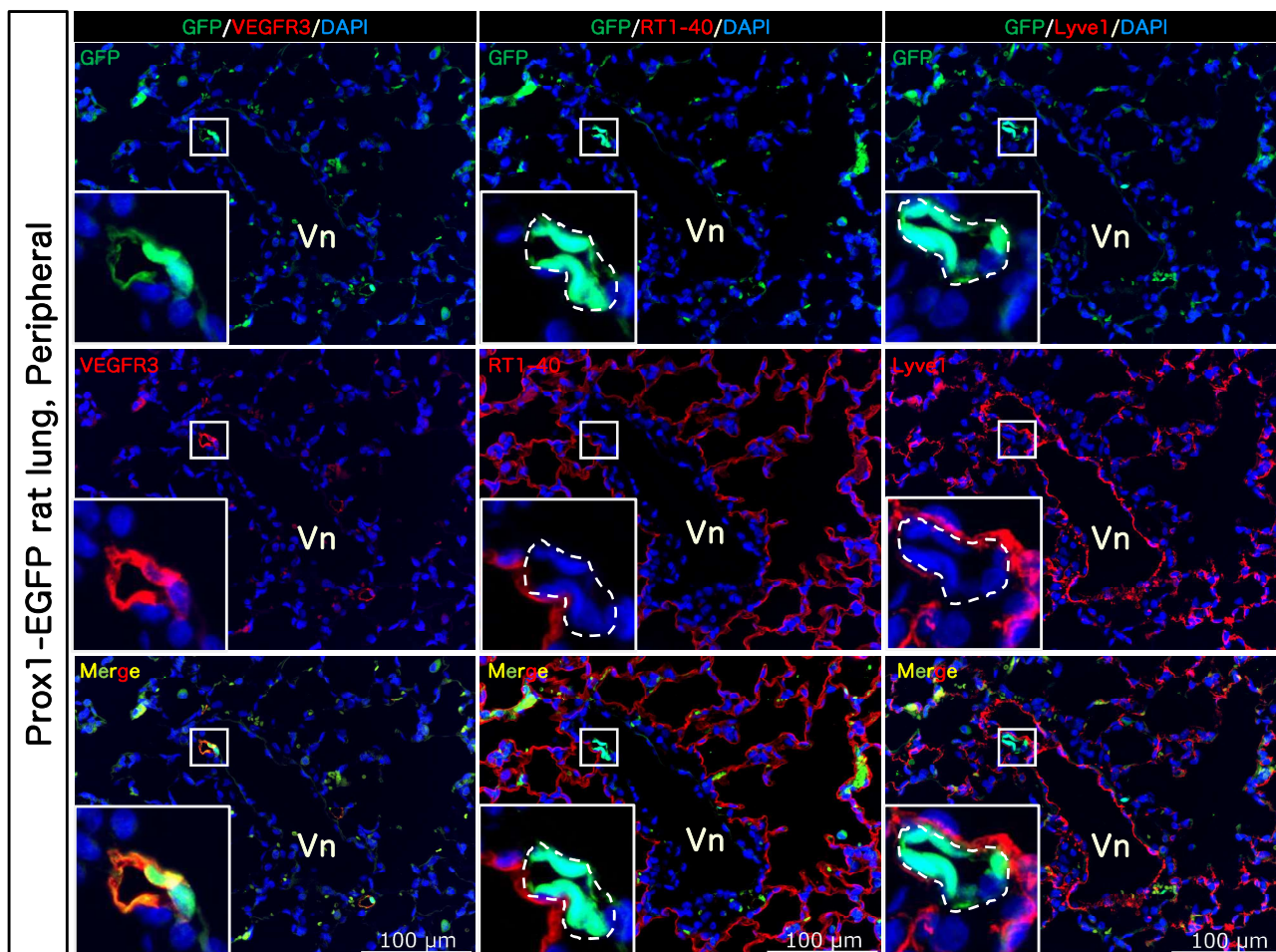

Supplement: Supplementary file 4 — Additional file 4: Fig. S4. Co-localization of vascular endothelial growth factor receptor 3 (VEGFR3) with lymphatic vessels in rat lungs. Lung sections from Prox1-EGFP transgenic rats were stained with three commercially available lymphatic markers, VEGFR3, lymphatic vessel endothelial hyaluronan receptor 1 (Lyve-1), and podoplanin (RT1-40), with co-staining of GFP and 4',6-diamidino-2-phenylindole (DAPI; a nucleus marker). Only VEGFR3 co-localized almost completely with GFP in the rat lungs, including in the peripheral regions. Br Bronchus and Vn Venule. [file 12989_2022_468_MOESM4_ESM.pdf]

Fig. S5

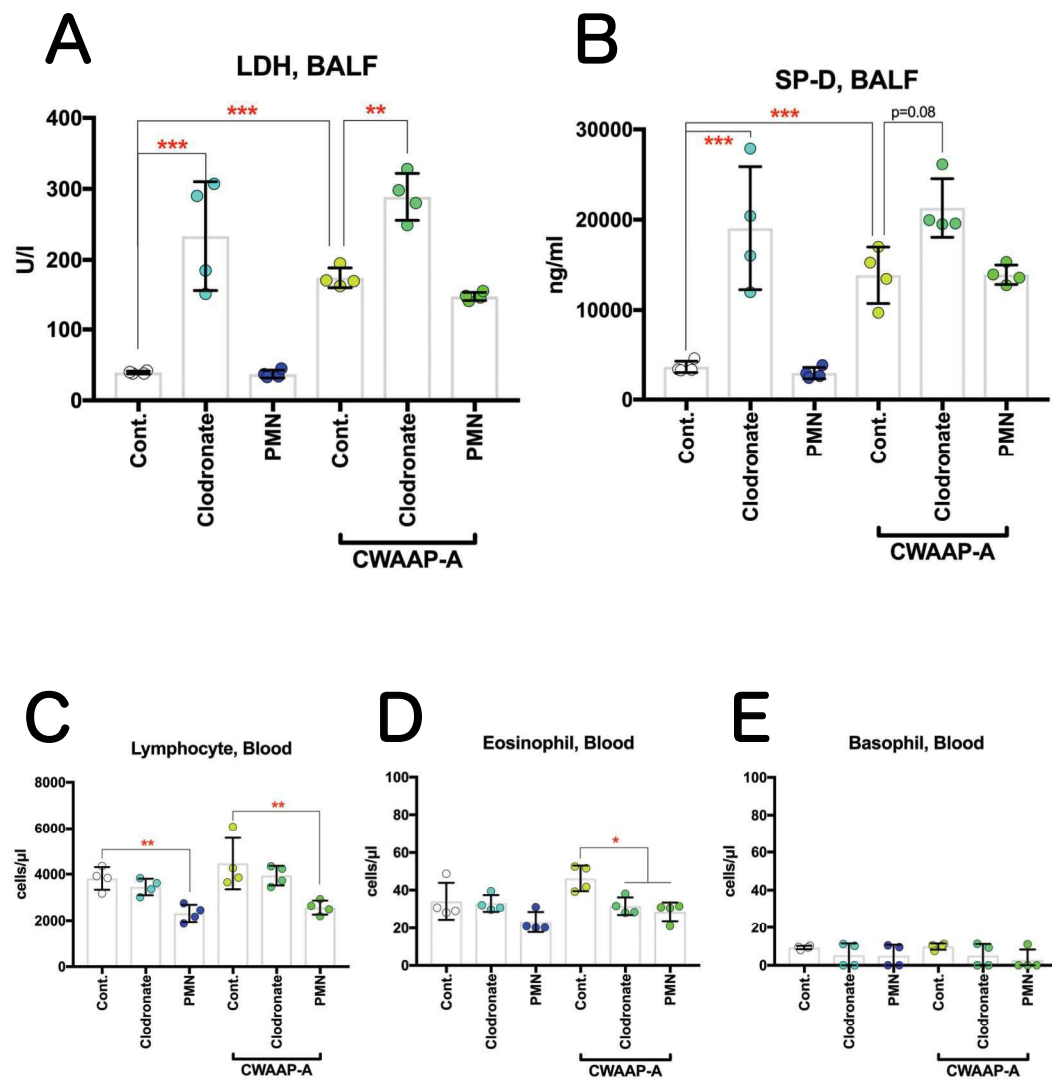

Supplement: Supplementary file 5 — Additional file 5: Fig. S5. The effect of pretreatment of the CWAAP-A-exposed rats with clodronate liposomes (to deplete macrophages) or polymorphonuclear leukocytes (PMN)-neutralizing antibodies (to deplete neutrophils) on BALF markers and cell populations. The lactate dehydrogenase (LDH) activity (A) and surfactant protein-D (SP-D) in the BALF B, and the number of lymphocytes (C), eosinophils (D) and basophils € in the plasma are shown. Tukey’s multiple comparison test: *p < 0.05, **p < 0.01, and ***p < 0.001, pairs indicated. [file 12989_2022_468_MOESM5_ESM.pdf]
