# Supplementary material for: Dose–response relationship of pulmonary disorders by inhalation exposure to cross-linked water-soluble acrylic acid polymers in F344 rats
Source: Part Fibre Toxicol. 2022 Apr 8;19:27. doi: 10.1186/s12989-022-00468-9 (PMC8994297; doi:10.1186/s12989-022-00468-9)
Supplement: Supplementary file 6 — Additional file 6: Fig. S6. Design of animal experimental protocols in this research. 13-week inhalation exposure study (A), and macrophages or neutrophils depletion study (B). [file 12989_2022_468_MOESM6_ESM.pdf]

Fig. S6

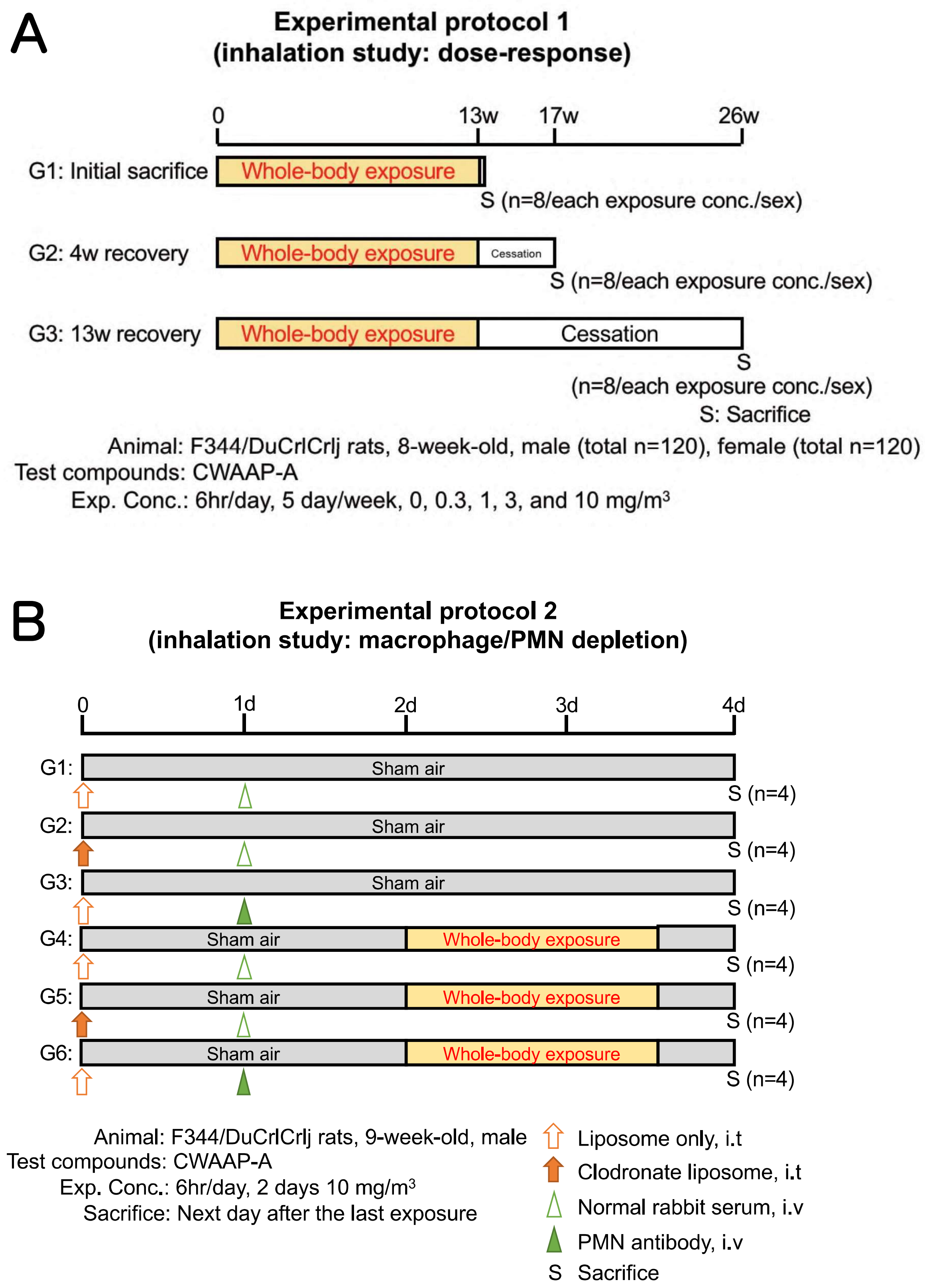
A
Experimental protocol 1
(inhalation study: dose-response)
0
13w
17w
26w
G1: Initial sacrifice
Whole-body exposure
S (n=8/each exposure conc./sex)
G2: 4w recovery
Whole-body exposure
Cessation
S (n=8/each exposure conc./sex)
G3: 13w recovery
Whole-body exposure
Cessation
S
(n=8/each exposure conc./sex)
S: Sacrifice
Animal: F344/DuCrlCrlj rats, 8-week-old, male (total n=120), female (total n=120)
Test compounds: CWAAP-A
Exp. Conc.: 6hr/day, 5 day/week, 0, 0.3, 1, 3, and 10 mg/m³
B
Experimental protocol 2
(inhalation study: macrophage/PMN depletion)
0
1d
2d
3d
4d
G1:
Sham air
S (n=4)
G2:
Sham air
S (n=4)
G3:
Sham air
S (n=4)
G4:
Sham air
Whole-body exposure
S (n=4)
G5:
Sham air
Whole-body exposure
S (n=4)
G6:
Sham air
Whole-body exposure
S (n=4)
Animal: F344/DuCrlCrlj rats, 9-week-old, male
Test compounds: CWAAP-A
Exp. Conc.: 6hr/day, 2 days 10 mg/m³
Sacrifice: Next day after the last exposure
Liposome only, i.t
Clodronate liposome, i.t
Normal rabbit serum, i.v
PMN antibody, i.v
S Sacrifice
